# Using Genome-Wide SNP Discovery and Genotyping to Reveal the Main Source of Population Differentiation in* Nothofagus dombeyi* (Mirb.) Oerst. in Chile

**DOI:** 10.1155/2016/3654093

**Published:** 2016-06-20

**Authors:** Rodrigo Hasbún, Jorge González, Carolina Iturra, Glenda Fuentes, Diego Alarcón, Eduardo Ruiz

**Affiliations:** ^1^Departamento de Silvicultura, Facultad de Ciencias Forestales, Universidad de Concepción, 4070386 Concepción, Chile; ^2^Departamento de Botánica, Facultad de Ciencias Naturales y Oceanográficas, Universidad de Concepción, 4070386 Concepción, Chile; ^3^Instituto de Ecología y Biodiversidad (IEB), Facultad de Ciencias, Universidad de Chile, 7800003 Santiago, Chile

## Abstract

Within a woody plant species, environmental heterogeneity has the potential to influence the distribution of genetic variation among populations through several evolutionary processes. In some species, a relationship between environmental characteristics and the distribution of genotypes can be detected, showing the importance of natural selection as the main source of differentiation.* Nothofagus dombeyi* (Mirb.) Oerst. (Nothofagaceae) is an endemic tree species occurring both in Chile and in Argentina temperate forests. Postglacial history has been studied with chloroplast DNA and evolutionary forces shaping genetic variation patterns have been analysed with isozymes but fine-scale genetic diversity studies are needed. The study of demographic and selection histories in* Nothofagus dombeyi* requires more informative markers such as single nucleotide polymorphisms (SNP). Genotyping-by-Sequencing tools now allow studying thousands of SNP markers at reasonable prices in nonmodel species. We investigated more than 10 K SNP loci for signatures of local adaptation and showed that interrogation of genomic resources can identify shifts in genetic diversity and putative adaptive signals in this nonmodel woody species.

## 1. Introduction

In population genetics and conservation the big question is genetic drift instead of natural selection. Definitely both processes determine evolution, but genetic drift operates randomly and depends on effective population size while natural selection proceeds nonrandomly and relies on environmental variables. The evolution towards hereditary adaptations to the current environment is determined by natural selection and has a direction; genetic drift instead is governed solely by chance. Consequently, drift acts on alleles, which generally have no phenotypic effect; instead selection favours certain alleles that increase fitness, reduce the unfavourable alleles frequencies, and ignore neutral alleles [1]. Knowing what the main microevolutionary force is is very relevant for rational genetic management of threatened species, especially for species with geographical distribution severely fragmented [2].

Approaches to addressing adaptive variation have been incorporated into the definition of evolutionary significant units [3, 4]. New technologies like Next Generation Sequencing (NGS) and fine-scale GIS, coupled with advances in computer hardware and software in the field of genomics [5–7], have allowed the development of new methods for comprehensive evaluation of adaptive diversity [8, 9]. The discovery and genotyping of massive genetic markers are now enabled by modern genomic tools at very low cost. This makes the study of adaptive genetic loci possible on a wide range of species, which can facilitate the identification of key biodiversity areas. Kirk and Freeland [10] reviewed some of the applications of neutral versus adaptive markers in molecular ecology, discussed some of the advantages that can be obtained by supplementing studies of molecular ecology with data from nonneutral molecular markers, and summarized new methods that allow generating data from loci under selection. Population genomic analyses require multilocus datasets from multiple populations and identification of nonneutral or adaptive loci by contrasting patterns of population divergence among genetic regions.

Studies in nonmodel organisms have shown relatively broad candidate genomic regions that are under selection, but it remains difficult to identify the genes (or the mutations) that are affected by selection. Increasing the density of markers in genome scans is paramount to overcome this problem, and validating signals of selection from particular genes using multiple methods should also help [4]. One of the most exciting developments in population genomics is the development of various reduced-representation protocols, collectively referred to as Genotyping-by-Sequencing (GBS), which allow sequencing of a subset of the genome through selective amplification of restriction fragments [10].

*Nothofagus dombeyi* (Mirb.) Oerst. (Nothofagaceae) is an endemic tree species occurring both in Chile and in Argentina temperate forests with a remarkably broad altitudinal and latitudinal distribution, across many different ecological gradients in the former [11]. The evergreen tree* N. dombeyi* is a pioneer species and constitutes an important element in the dynamics of South American forest. Its postglacial history has been studied with chloroplast DNA and evolutionary forces shaping genetic variation patterns have been analysed with isozymes [12, 13]. However, genome-wide scan methods using thousands of markers to study a representative portion of the genome are needed.

In this work, we assess GBS in the nonmodel woody species* N. dombeyi* to develop high quantity of informative markers such as single nucleotide polymorphisms (SNP). Our aim is to determine the contribution of selection and molecular adaptation to shaping genome-wide variation. We expect higher genetic population differentiation (for ecological separated localities) for adaptive SNP than neutral SNP if natural selection is the principal source of differentiation. Alternatively, if other sources of differentiation (mutation, genetic drift, and migration) are relevant, they will equally affect both types of SNP. Knowing the contribution of selection effect to shaping genome variation patterns will have many applications for biodiversity conservation, especially in endangered species, because neutral and adaptive genetic diversity will likely have different impacts on long-term survival. In fact, in most cases, only adaptive diversity will allow a population to adapt to changing environmental conditions.

## 2. Materials and Methods

### 2.1. Sampling Design

#### 2.1.1. Niche Modelling

To consider the remarkably broad altitudinal and latitudinal distribution of this species, across many different ecological gradients, we used the method proposed by Alarcón and Cavieres [14] to niche modelling of* Nothofagus dombeyi* in Chile using BIOMOD [15]. Eight variables were selected from the WorldClim global climate database [16] corresponding to the present climate conditions with a 30-arc-second grid resolution, with the least correlation among them for the studied species range area. Four variables were related to energy constraints: (a) BIO2: mean diurnal temperature range; (b) BIO4: temperature seasonality; (c) BIO5: maximum temperature in the warmest month; and (d) BIO6: minimum temperature in the coldest month. Other four variables were related to water availability: (e) BIO12: annual precipitation; (f) BIO15: precipitation seasonality; (g) BIO18: precipitation in the warmest quarter; and (h) BIO19: precipitation in the coldest quarter. Then, we projected the current and future distribution (year 2050) considering a conservative future climate projection CSIRO B2A 2050 by using the tools of BIOMOD software. Further, we identified geographical areas with potential habitat loss, which should be identified as high priority in genetic conservation programs.

#### 2.1.2. Ecological Regions or Strata

Relatively homogeneous units in ecological terms (strata) were defined from natural populations of the species associated with the geographical areas projected in its ecological niche modelling. The Calinski-Harabasz criterion, which is a pseudo-*F* statistic as in ANOVA, was used to assess the best number of strata identified by *K*-means partitioning [17].

### 2.2. DNA Sample and Library Preparation

#### 2.2.1. Plant Material and Genomic DNA Isolation

Adult trees with a diameter higher than 50 cm were sampled during the growing season of 2013-2014 from twenty-one sites covering almost the entire range of the species* N. dombeyi* in Chile. One to four sites were assigned to each stratum according to its superficies and 2 to 9 samples per site were taken (Table 1; Supplementary Figure 1 in Supplementary Material available online at http://dx.doi.org/10.1155/2016/3654093).

DNA extraction was performed using a Qiagen DNeasy Plant kit (Qiagen Inc., USA). Lyophilized leaf tissue (20 mg) was ground in a Precellys®24 (Precellys, USA) homogenizer with two 1/4′′ ceramic spheres (MP BIOMEDICALS, USA) and AP1 buffer. The objective is grinding tissues and lysing cells prior to DNA extraction. DNA extraction protocol was done following the manufacturer instructions but elution was done with 30 *μ*L (instead of 100 *μ*L) to increase the final DNA concentration in the eluate (>100 ng/*μ*L). The integrity of genomic DNA was evaluated by agarose gel and quantified using a Qubit fluorometer (Invitrogen, USA).

#### 2.2.2. Library Preparation and High-Throughput Sequencing

Library preparation and high-throughput sequencing were performed at University of Wisconsin Biotechnology Center (DNA Sequencing Facility). The GBS genomic library preparation was done following the protocol detailed by Elshire et al. [18] with the methylation-sensitive restriction enzyme ApeKI and 96 custom barcodes. Illumina high-throughput sequencing was conducted on an Illumina HiSeq 2000 (Illumina, USA) using 100 bp single-end sequencing runs. The samples were sequenced across one Illumina lane. Base calling was performed in Casava v1.8.2 (Illumina, USA).

### 2.3. Nonreference SNP Calling

#### 2.3.1. De Novo Identification of Loci/Alleles

Sequence results were analysed and SNP genotypes were assigned using the UNEAK (Universal Network Enabled Analysis Kit) GBS pipeline [19], which is part of the TASSEL 3.0 [20] bioinformatic analysis package. This pipeline does not depend on a reference sequence, which is the actual case for* N*.* dombeyi*. SNP discovery is performed directly within pairs of matched sequence tags (unique sequence representing a group of reads) and filtered through network analysis. The network filter trimmed reads to 64 bp to reduce the effects of error sequencing and enabled efficient storage of data in bit format. SNP were assigned with default settings. Briefly, tags differing by a single nucleotide were retained as SNP and those with a minor allele frequency 0.05 were removed to minimize the impact of sequencing errors [19]. We used a minimum call rate of 0 and additional filters were applied in next steps.

#### 2.3.2. Post-SNP Calling Filters and Imputation

Given that we are trying to find SNP for population genetic analysis, we applied some filters to remove loci and individuals that contain very low levels of information prior to further analysis. We applied two functions of TASSEL 5.2 that removed all SNP (rows) and then samples (columns) containing 90% or more “*N*” values (indicating that neither allele is designated). These *N*s represent individuals where the allele cannot be called from the sequence reads. This is because either no read is available at this site (for this individual) or the sequence quality is too low to be called.

In order to cope with missing data, genotype imputation was used to fill in the missing data and improve the power of downstream analyses. We used LinkImpute implemented in TASSEL 5.2, a software package based on a *k*-nearest neighbour genotype imputation method, LD-*k*NNi [21]. This imputation method was designed specifically for nonmodel organisms in which genomic resources are poorly developed and marker order is unreliable or unknown.

### 2.4. Detection of Selection Footprints

To identify adaptive SNP (putative loci under selection), we used LOSITAN [22]. LOSITAN is a selection detection workbench based on the Fst-outlier methods. We used 50,000 simulations, 0.99 for confidence interval, false discovery rate of 0.05, mutation model “Infinite Alleles,” and the options “Neutral mean Fst” and “Force mean Fst”, which iteratively identify and remove Fst outliers when calculating the global distribution of Fst. Our interest is in patterns of adaptation driven by environmental gradients; therefore we focused on outlier patterns indicating divergent selection (Fst significantly higher than neutral expectations).

LOSITAN analyses were complemented with BayeScan 2.1 [23] for estimating the posterior probability that a given locus is affected by selection. Briefly, prior odds of 100 were used for identifying the top candidates of the selected loci and a total of 50,000 reversible-jump Markov Chain Monte Carlo chains were run with a thinning interval of 10, following 20 pilot runs of 5,000 iterations each, and a burn-in length of 50,000. Loci were considered outliers with an FDR of 0.05.

To confirm the adaptive SNP detected by previous methods, the spatial analysis method (SAM) implemented in the program Sam*β*ada v0.5.1 [24] was used. We conducted the analysis using the 10,109 SNP detected for* N. dombeyi* in Chile. Sam*β*ada uses logistic regressions to model the probability of presence of an allelic variant in a polymorphic marker given the environmental conditions of the sampling locations. Eight environmental variables previously described in Section 2.1.1 were used (temperature related → BIO2, BIO4, BIO5, and BIO6; precipitation related → BIO12, BIO15, BIO18, and BIO19). Regarding genotypes, each of the states of a given SNP is considered independently as binary presence/absence in each sample. Our biallelic SNP were recoded as three distinct genotypes (AA, AB, and BB). A maximum likelihood approach is used to fit the models using univariate analyses. Each model for a given genotype is compared to a constant model, where the probability of presence of the genotype is the same at each location. The statistical significance threshold was set to 1% before applying Bonferroni correction. Significance was assessed with log likelihood ratio (*G*) tests [25] selecting loci/allele that tested higher than the 99th percentile of the *G* score distribution.

### 2.5. Estimation of Genome-Wide Genetic Variation and Differentiation

We made all estimations in parallel with neutral SNP (10,109) and adaptive SNP.

#### 2.5.1. Basic Statistics of Genetic Variation

All the results were obtained using the adegenet [26, 27] and hierFstat [28] R packages. Basic statistics were estimated including observed heterozygosity (Ho) and genetic diversity (Hs) within population. Also, overall gene diversity (Ht) and corrected Ht (Htp), gene diversity among samples (Dst), and corrected Dst (Dstp) were estimated. Fst and corrected Fst (Fstp) were assessed as well as Fis following Nei [29] per overall loci. Dest, a measure of population differentiation as defined by Jost [30], was also calculated. The degree of genetic differentiation among populations is expected to be low for neutral SNP but highly divergent in SNP subject to directional selection.

#### 2.5.2. Population Structure

To describe the genetic biodiversity of a species, more important than diversity among individuals is the diversity between groups of individuals. First we analysed individual data to identify populations, or more large genetic clusters, and then we described these clusters by adegenet R package. To get a simplified picture of the genetic diversity observed among individuals or populations we used Principal Component Analysis (PCA). Discriminant Analysis of Principal Components (DAPC) function was used to describe the relationships between these clusters. The main results of DAPC were DAPC scatterplots.

For each pair of strata, we computed pairwise Fst values with hierFstat R package. Principal Coordinates Analysis (PCoA) on Fst values was performed to detect major genetic clusters (*K*) at individual level.

#### 2.5.3. Detecting Locus Contributions

In DAPC, the variables actually analysed are principal components of a PCA. Loadings of these variables are generally uninformative, since PCs themselves do not all have straightforward interpretations. However, we can also compute contributions of the alleles, which can turn out to be very informative. In general, there are many alleles and their contribution is best plotted for a single discriminant function at a time using adegenet R package.

## 3. Results and Discussion

### 3.1. Niche Modelling and Strata Definition

**Figure 1:**
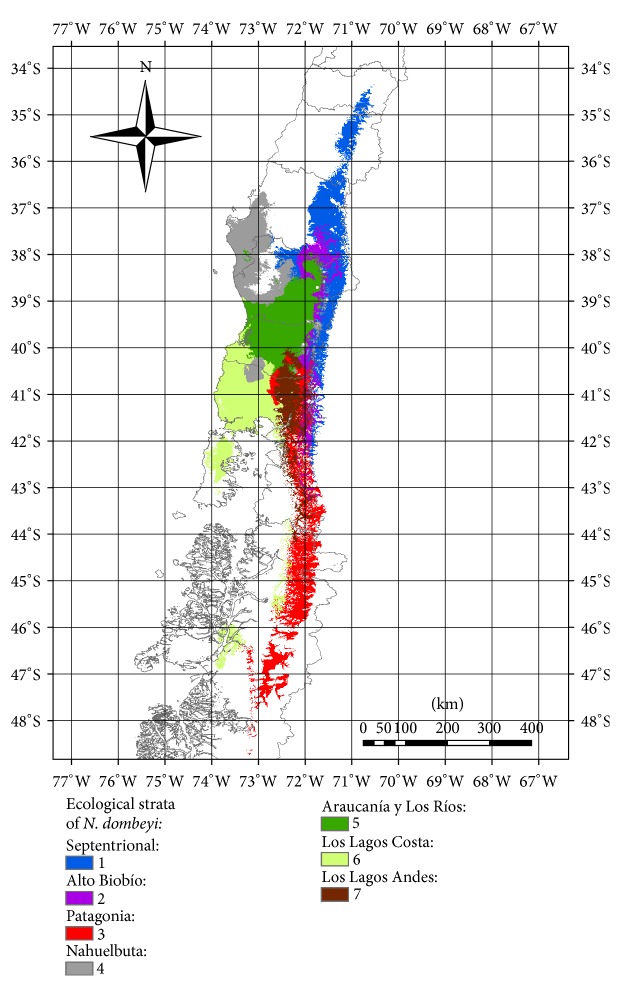
Using ecological niche models to identify different strata for* Nothofagus dombeyi* in Chile.

Modelled area of* N. dombeyi* presence under present climate conditions is 88,174 km^2^ and in the future (year 2050) under dispersal constraints is 72,928 km^2^. Although it is not qualified with a status of threatened species, we estimated a decrease of almost 20% of its habitat area in a relatively short time, particularly in the northern populations, associated with Mediterranean-influenced climate, which is at least worrisome considering we used the most conservative future climate scenario. Fuss et al. [31] showed that the actual reality is the worst climate scenario projected. The *K*-means analyses, applied to four best least correlated variables of WorldClim (BIO2, BIO4, BIO12, and BIO15), identified seven strata groups of* N. dombeyi* in Chile at very broad spatial scales (Figure 1). These strata represented relatively homogeneous habitats according to the climatic key variables mentioned. The strata named Septentrional (#1), Alto Biobío (#2), Los Lagos Andes (#7), and Patagonia (#3) form the latitudinal gradient across Andes Mountain range including higher altitudes, while the strata Nahuelbuta (#4), Araucanía y Los Ríos (#5), and Los Lagos Costa (#6) present some oceanic influence at a gradient located in Coastal Mountain range and central valley of the central distribution of* N. dombeyi* at lower elevation. The strata were coherently identified as the bioclimatic transition from temperate biome with some Mediterranean influence, especially in the northern portion of the Septentrional stratum and then the typical temperate bioclimatic classification across the rest of the distribution of the species [32].

### 3.2. Assessment of* N. dombeyi* Genotypes Sampled by GBS Using* Ape*KI

We obtained more than 17 Gb out of 172,171,356 reads, from which 85.82% keep a score *Q* ≥ 30. From the 96 barcoded samples in our study, an average of 1.5 million (SD 1.7 million) sequence reads per sample were obtained (range from 278 to 8.2 millions) (see Supplementary Figure 2). After removal of SNP with a minor allele frequency 0.05 and removing samples and SNP with more than 90% “NN” (unassigned) genotypes, the dataset consisted of 73 individuals with 10,109 binary SNP from seven strata. This dataset was subjected to the impute step and finally total numbers of “NN” genotypes were 19,067 (0.03%) missing data.

### 3.3. Putative under Selection SNP (Adaptive SNP)

Using the LOSITAN approach, 124 adaptive SNP (1.2%) were identified (directionally selected) at the false discovery rate (FDR) threshold of 0.1 (Table 2; Supplementary Table 1). We detected 99 adaptive SNP in BayeScan and there were 48 overlap cases between the loci reported by each method (see Supplementary Table 1). The adaptive SNP detected by both approaches were limited (39%), possibly reflecting discrepancies in their methodologies. However, all the 124 SNP identified by LOSITAN had Fst values ≥ 0.30 (estimated across the 73* N. dombeyi* individuals and 10,109 loci), which may be considered as strongly differentiated.

Using Sam*β*ada we detected 2,406 significant genotypes associated with a given environmental variable. They represent 884 SNP (8.7% of total SNP assessed) and only 3% correspond to heterozygous genotypes. From the 124 outlier SNP identified by LOSITAN, genotypes in 121 SNP were identified as associated with environmental variables by Sam*β*ada (see Supplementary Table 1). Genetic differentiation associated with both temperature and precipitation gradients was detected.

### 3.4. Genome-Wide Genetic Variation and Differentiation

**Figure 2:**
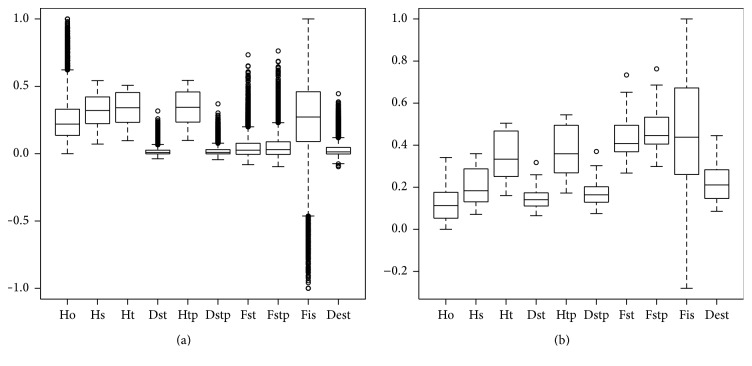
Summary statistics of genetic variation existing in* Nothofagus dombeyi* in Chile estimated by 10,109 neutral SNP (a) or 124 adaptive SNP (b). Ho: heterozygosity within population; Hs: genetic diversity within population; Ht: overall gene diversity; Http: corrected Ht; Dst: gene diversity among samples; Dstp: corrected Dst; Fst: fixation index; Fstp: corrected Fst; Fis: inbreeding coefficient per overall loci; Dest: measure of population differentiation.

Using neutral plus outlier SNP (10,109), different basic statistics of genetic variation of* N. dombeyi* in Chile show low to medium genetic diversity level and low level of strata differentiation (Figure 2). The low genetic structure found indicates relatively high gene flow, which is consistent with the fact that* N. dombeyi* has a distribution more or less continuous and is wind pollinated. This result is similar to other results obtained with the same and other species of* Nothofagus* gender [33, 34]. With outlier SNP, the indicators based on heterozygosity increase and *F* statistics shows clear differences within strata.

Overall average pairwise Fst values among populations, calculated over the set of the 10,109 SNP, show medium population structuring across the distribution of* N. dombeyi* in Chile (Table 3). The average pairwise Fst values ranged from 0.062 between two very close strata (4 and 5) in the Coastal Mountains to 0.258 between strata 1 and 3, both in Andean Mountains but in the extremes of distribution (Table 1). The Principal Coordinates Analysis (PCoA) plot revealed a geographically ordered pattern (see Supplementary Figure 3). The first PC suggests the existence of two clades in the data, while the second one shows groups of closely related isolates arranged along a cline of genetic differentiation. Premoli [35] found continuous clinal genetic variation in populations of* N. pumilio* along the altitudinal gradient, as a result of adaptive responses to ecological gradients and/or restrictions for gene flow. The same pattern is shown by neutral SNP and adaptive SNP but with a more clear delimitation in the latter case. This structure was confirmed by a neighbour-joining (NJ) tree (Supplementary Figure 4). Again higher resolution is achieved with adaptive SNP. As expected, both approaches give congruent results, but both are complementary; NJ shows bunches of related individuals, but the cline of genetic differentiation is much clearer in PCA.

**Figure 3:**
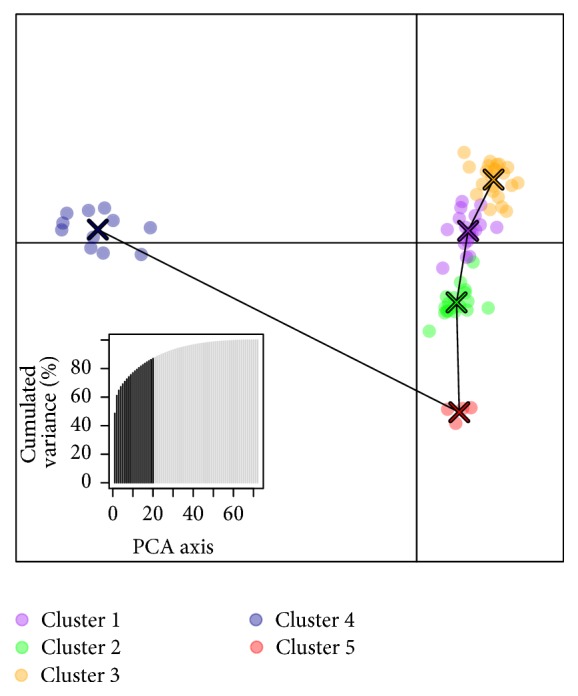
Discriminant Analysis of Principal Components (DAPC) scatterplot drawn using 124 outlier single nucleotide polymorphisms (SNP) across 73* Nothofagus dombeyi* individuals in the R package adegenet. Dots represent individuals, with colours denoting cluster allocation. Percentages of cumulated variance explained by Principal Component 1 (PC1) to PC20 are shown in the bottom left corner. Minimum spanning tree based on the (squared) distances between clusters within the entire space is shown.

Visualisation of broad-scale population structure using a DAPC (Figure 3 and Table 4) with 124 outlier SNP revealed two distinct genetic groups. One group included individuals sampled in all strata corresponding to Clusters 1, 2, 3, and 5 (Table 4), and the other group comprised only ten individuals of stratum 3 plus one individual of stratum 2 forming Cluster 4 (represented almost exclusively by individuals of Patagonia stratum). Also the actual proximities between clusters show the great genetic distance of Cluster 4 with the rest. This result is consistent with a north–south phylogenetic divergence at* c*. 43°S found within subgenus* Nothofagus* in southern South America, including* N. dombeyi* [12, 36].

Clearly, when examining patterns of* N. dombeyi* population differentiation at neutral versus adaptive SNP, we have detected several distinct differences. Primarily stochastic processes drive differentiation of neutral SNP, whereas both selective and stochastic processes drive that of adaptive SNP, for example, [37–39]. This result supports the hypothesis that putative under selection SNP are being affected by adaptation, which is not affecting the neutral SNP. The next step is to characterize the phenotypic outcomes of alternative genotypes to confirm if the mechanism is genetic drift or selection, similar to previous studies in* Nothofagus pumilio* [40, 41]. Ideally, phenotype-genotype association studies should be followed by the profiling of gene expression, functional tests, and selection tests to determine that a gene or genes are involved in shaping an adaptive trait [42].

### 3.5. Loci Contributions

**Figure 4:**
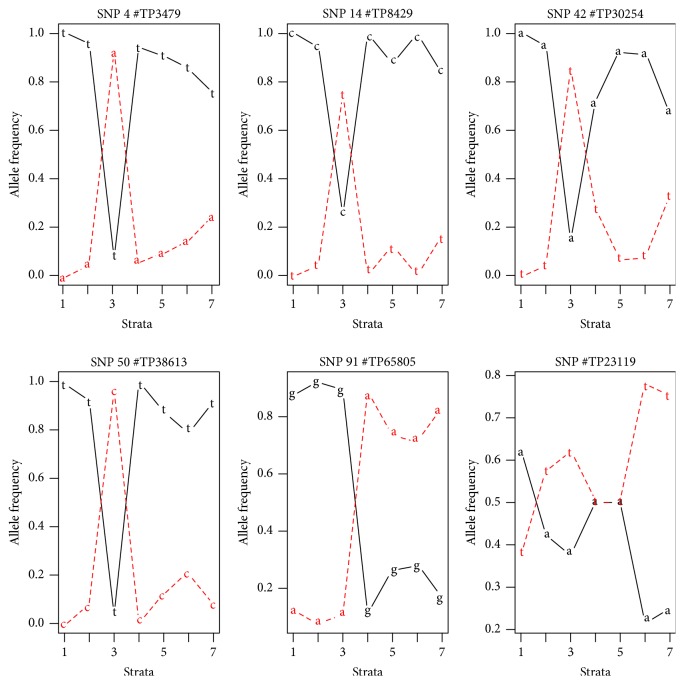
Spatial distributions of single nucleotide polymorphism (SNP) loci/genotypes in* N. dombeyi* in Chile for each strata. The graphs show frequencies of loci/genotypes differentiating among strata (SNP #3479, SNP #8429, SNP #30254, SNP #38613, and SNP #65805) and no differentiating among strata (SNP #23119).

Over 24% of the adaptive SNP detected by LOSITAN had a high loading (here defined as ≥0.02) in one or two of the three PC in a PCA with more than 10 K loci of the Chilean population of* N. dombeyi* (Supplementary Figures 5, 6, and 7), thus confirming its contribution to population structure. Adaptive SNP ID 4, 14, 42, 50, and 91 are five of the most discriminating loci and their allele frequencies over seven strata show a high or even dominant allele frequency in stratum 3 (Figure 4). This is the southernmost stratum (#3 Patagonia) and this may justify the differences in allele frequencies. Irrespective of the mechanism underlying these changes (drift or selection), this illustrates that, in the natural distribution of* N. dombeyi* in Chile, specific nucleotides can undergo drastic changes within only a hundred kilometres of distance. Our results exhibited spatial patterns differentiating one stratum in loci which could occur in genomic regions or genes for important functions of* N. dombeyi* across their habitat. For example, the GBS sequence tags of adaptive SNP ID 43 (TP30852) show a high identity with a heat shock protein or adaptive SNP ID 44 (TP32296) with histone acetyltransferase enzyme (data not shown). The first is fundamental for heat or other environmental stresses and the second can be involved in drought sensing or another response to environmental factors. We therefore suggest that these two SNP may be interesting candidates for future functional studies that might facilitate other studies, for example, [43] focused on the development of genomic resources for* Nothofagus* species. However, improvements in study design and analyses of replicated studies will be needed before this very promising approach can be brought to application for managing genetic resources [44].

## 4. Conclusions

Development of 10,109 genome-wide SNP for* N. dombeyi* using GBS made evaluation of genomic diversity and fine-scale population structure possible for the first time in this species in Chile. Results showed that genome-wide patterns of genetic diversity and differentiation varied widely across the genome. As such, we identified numerous genomic regions exhibiting signatures of divergent selection. We have also provided strong evidence of substantial genetic differentiation associated with both temperature and precipitation gradients, suggesting local adaptation.

## Supplementary Material

The supplementary material contain information of distribution of sampling locations (Figure S1), and the read number by sample after demultiplexing step in GBS process (Figure S2). The supplementary information also contains the complete list of outlier SNP as putative candidates for adaptation and visualization of genetic relationships among samples by PCA (Figure S3) and neighbour-joining (NJ) tree (Figure S4). Lastly, the loadings of outliers SNPs showing the most contributing loci to PC1, PC2 y PC3 are given in Figure S5, S6 and S7, respectively.

## Figures and Tables

**Table 1:** Strata code and location, geographical coordinates, and sample size of the sampled individuals of *Nothofagus dombeyi* in Chile.

Stratum	Location (code)	Latitude	Longitude	*N*
1	Altos Lircay (AL)	−35.599162	−71.044414	4
1	Antuco (AN)	−37.343457	−71.615626	5

2	Ralco (RA)	−37.925041	−71.575168	6
2	Termas de Tolhuaca (TT)	−38.235047	−71.727552	9

3	Lago La Paloma (LP)	−45.876213	−72.070813	4
3	El Machi (EM)	−45.009553	−71.906872	5
3	Near Villa Amengual (NVAM)	−45.008498	−71.908366	2
3	Villa Amengual (VAM)	−45.007328	−71.911175	5

4	Nonguén (NO)	−36.879745	−72.987923	5
4	Villa Las Araucarias (VA)	−36.879774	−72.987981	6
4	Caramávida (CA)	−36.879774	−72.987981	4

5	Mariquina (MA)	−39.471811	−73.055799	4
5	Lago Riñihue (LRI)	−39.480193	−73.048809	3
5	Fundo Llancahue (FL)	−39.858686	−73.141572	3
5	Lago Neltume (LN)	−39.859282	−73.141650	3
5	Parque Nacional Villarrica (PNV)	−39.341073	−71.972351	5
5	Loncoche Interior (LI)	−39.341820	−71.972232	3
5	Melipeuco (ME)	−38.912949	−71.704088	3

6	Las Trancas (LT)	−40.221101	−73.362430	5
6	Camino Osorno a Maicolpué (COM)	−40.598321	−73.497015	4

7	Parque Nacional Puyehue (PNP)	−40.737218	−72.306050	3
7	Lago Rupanco (LR)	−40.736785	−72.302922	5

**Table 2:** Representative list of outlier single nucleotide polymorphisms (SNP) as putative candidates for adaptation in *Nothofagus dombeyi* in Chile and their significant associations with environmental variables using Sam*β*ada. Italic values show an SNP that is not a significant outlier.

Locus	SNP ID	LOSITAN			BayeScan	Sam*β*ada							
		Het	Fst	*P* (Simul Fst < sample Fst)		BIO2	BIO4	BIO5	BIO6	BIO12	BIO15	BIO18	BIO19
TP3479	4	0.368	0.545	0.99999	Yes	x		x			x		x
TP8429	14	0.282	0.496	0.99996	—	x		x			x		x
TP23119	—	0.491	0.035	*0.46921*									
TP30254	42	0.385	0.442	0.99996	Yes	x		x			x		x
TP30852	43	0.438	0.438	1.00000	Yes	x		x			x		x
TP32296	44	0.467	0.377	0.99991	Yes	x		x			x	x	
TP38613	50	0.364	0.618	1.00000	Yes	x		x			x		x
TP65805	91	0.540	0.488	1.00000	Yes		x		x				

**Table 3:** Average pairwise Fst among seven strata with *Nothofagus dombeyi* presence in Chile based on a set of 10,109 single nucleotide polymorphisms (SNP). Fst 0.15–0.2 are moderately differentiated in bold font and Fst > 0.25 are considered strongly differentiated in bold and italic font.

Strata	1	2	3	4	5	6
2	0.074					
3	***0.258***	**0.193**				
4	0.111	0.074	**0.183**			
5	0.109	0.066	0.138	0.062		
6	**0.156**	0.108	0.140	0.096	0.069	
7	**0.172**	0.121	0.141	0.110	0.081	0.096

**Table 4:** Individual allocation to five genetic clusters according to strata determined by niche modelling of *N. dombeyi* in Chile. Clusters were identified by find.cluster function of adegenet R package using 124 adaptive single nucleotide polymorphisms (SNP).

Strata	Cluster assignation				
	C1	C2	C3	C4	C5
1	—	—	8	—	—
2	—	—	12	1	—
3	—	—	—	10	4
4	8	—	1	—	—
5	10	5	1	—	—
6	—	7	—	—	—
7	—	6	—	—	—
