# Supplementary material for: Using Genome-Wide SNP Discovery and Genotyping to Reveal the Main Source of Population Differentiation in Nothofagus dombeyi (Mirb.) Oerst. in Chile
Source: Int J Genomics. 2016 Jun 20;2016:3654093. doi: 10.1155/2016/3654093 (PMC4944027; doi:10.1155/2016/3654093)
Supplement: Supplementary file 1 — The supplementary material contain information of distribution of sampling locations (Figure S1), and the read number by sample after demultiplexing step in GBS process (Figure S2). The supplementary information also contains the complete list of outlier SNP as putative candidates for adaptation and visualization of genetic relationships among samples by PCA (Figure S3) and neighbour-joining (NJ) tree (Figure S4). Lastly, the loadings of outliers SNPs showing the most contributing loci to PC1, PC2 y PC3 are given in Figure S5, S6 and S7, respectively. [file 3654093.f1.docx]

**Supplementary Information**


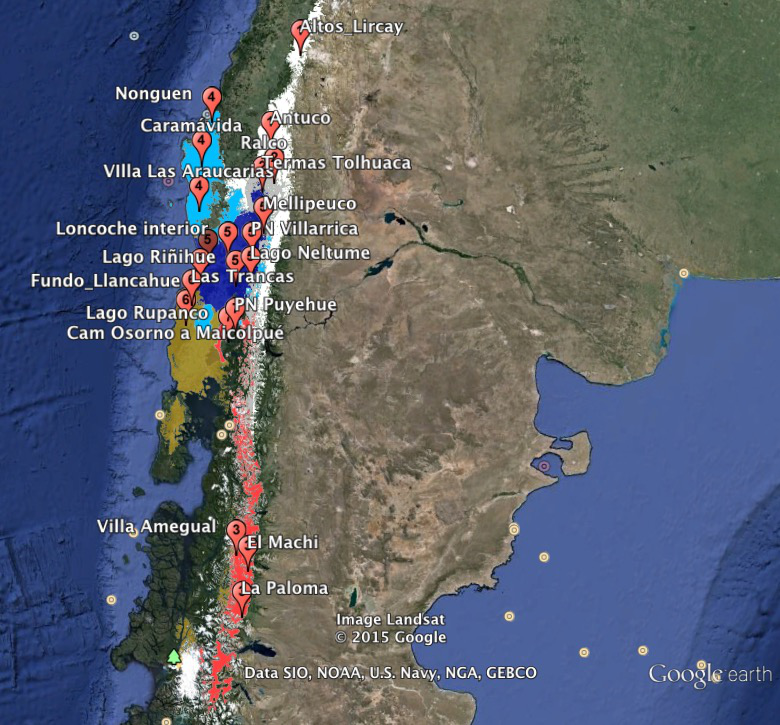


Supplementary Figure 1. Distribution of sampling locations according to size of strata of *N. dombeyi* in Chile. White represent strata 1, grey strata 2, red strata 3, light blue strata 4, blue strata 5, yellow strata 6 and green strata 7.

Supplementary Figure 2. Read number by sample after demultiplexing step in 96 barcoded genotyping-by-sequencing (GBS) library of *N. dombeyi* sequencing by Illumina HiSeq 2000 using 100 bp single-end sequencing runs.

[[

Supplementary Table 1. List of outlier single nucleotide polymorphism (SNP) as putative candidates for adaptation in *N. dombeyi* in Chile and their significant associations with environmental variables using Samβada. Grey boxes reflect SNPs that is not significant outlier.

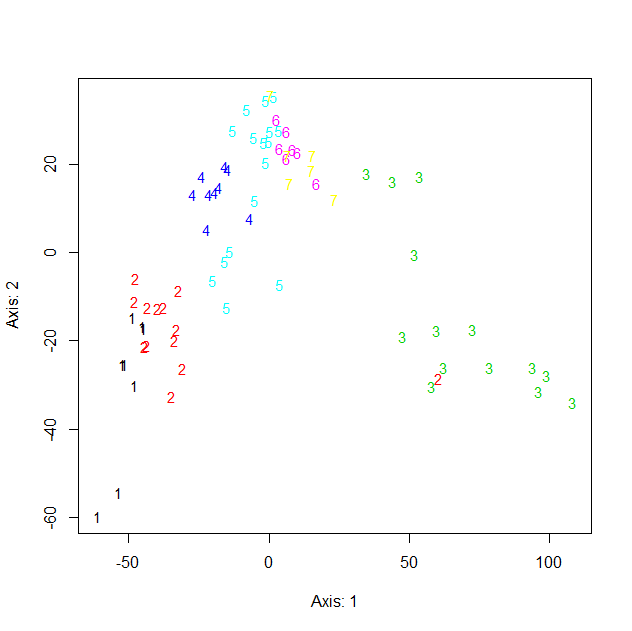


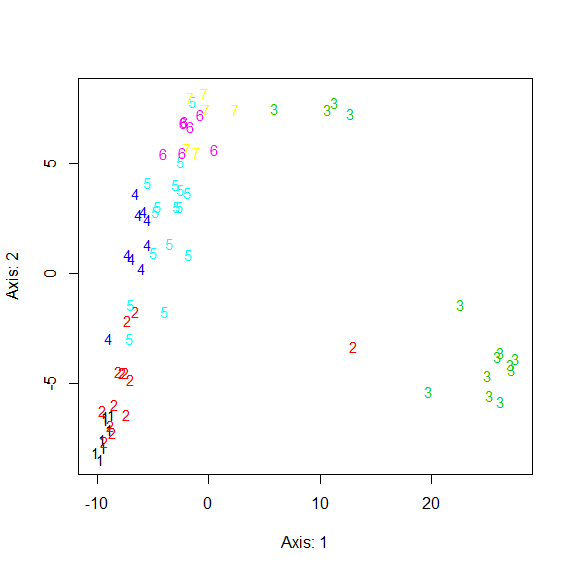


Supplementary Figure 3. Principal Component Analysis (PCA) using all (10,109) single nucleotide polymorphism (SNP) (upper box) or only outliers SNP (124) (lower box) detected in *N. dombeyi* in Chile (N=73). Scatterplot draw using hierfstat based in the principal components (PCs) individual pairwaise Fst. Coloured based on geographic origins . Numbers represent the strata of individual origin (Table 1).


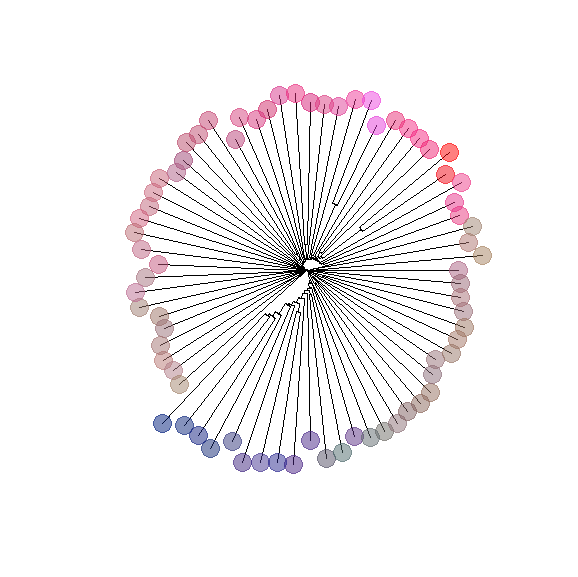


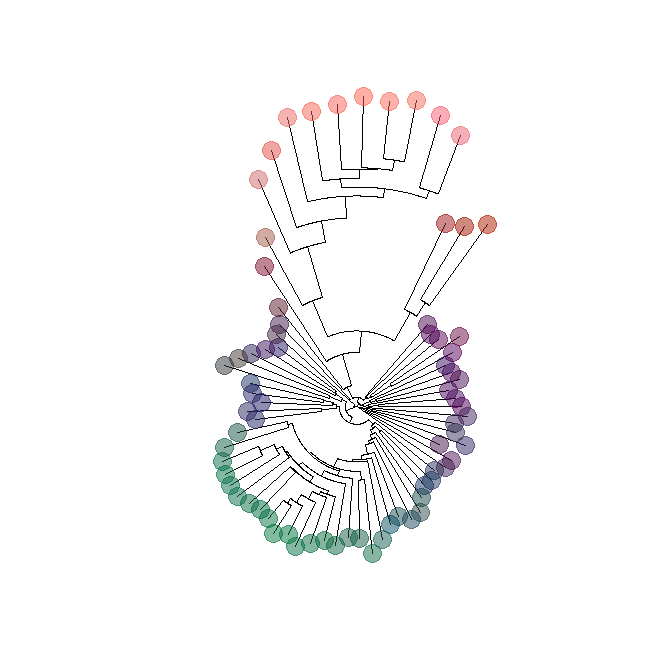


Supplementary Figure 4. Unrooted neighbour-joining (NJ) tree showing colour genotype separation in N. dombeyi in Chile (N=73) based on all (10,109) single nucleotide polymorphism (SNP) (upper NJ) or only outliers SNP (124) (lower NJ). Tree drawn in adegenet using pairwise distances between individuals. The genetic diversity is represented in two complementary ways: by the distances (further away = more genetically different), and by the colors (more different colors = more genetically different).


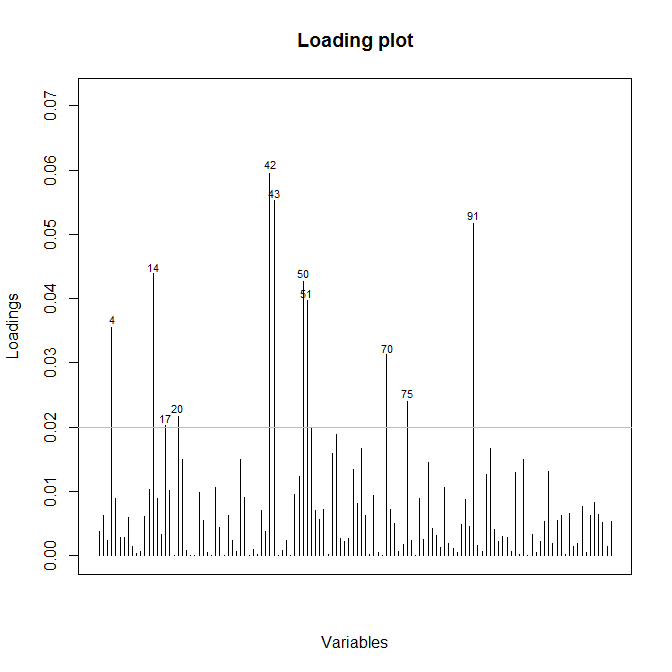


Supplementary Figure 5. Genetic loads (Loadings) of outliers single nucleotide polymorphism (SNP) showing the most contributing loci to PC1.

i


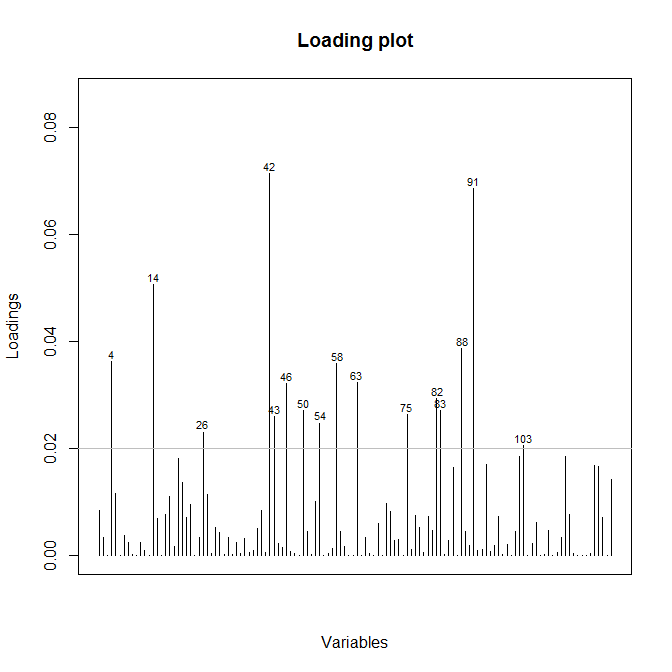


Supplementary Figure 6. Genetic loads (Loadings) of outliers single nucleotide polymorphism (SNP) showing the most contributing loci to PC2. Numbers correspond to SNP ID (Supplem. Table 1)


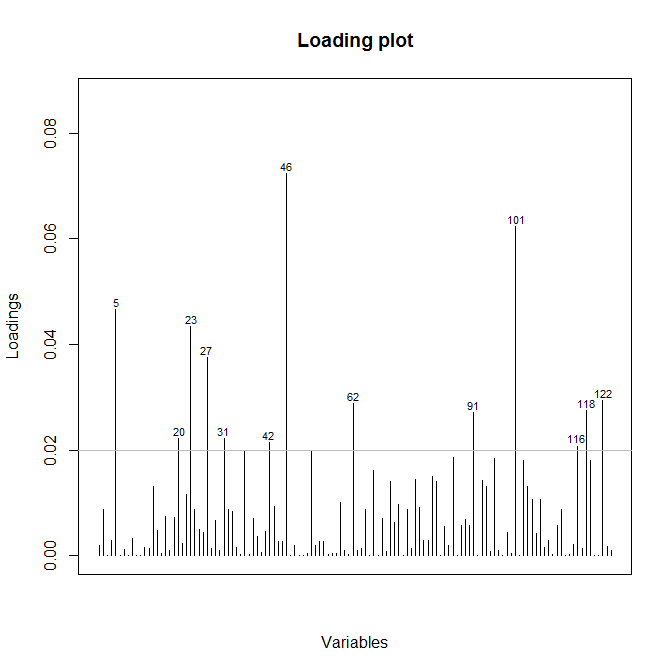


Supplementary Figure 7. Genetic loads (Loadings) of outliers single nucleotide polymorphism (SNP) showing the most contributing loci to PC3.
